# Challenges in transitioning from adolescent to Adult Mental Health Services for young adults with ADHD in Italy: an observational study

**DOI:** 10.1017/S2045796024000544

**Published:** 2024-10-24

**Authors:** Elisa Roberti, Antonio Clavenna, Eleonora Basso, Carmela Bravaccio, Maria Pia Riccio, Maurizio Pincherle, Maddalena Duca, Claudia Giordani, Francesca Scarpellini, Rita Campi, Michele Giardino, Michele Zanetti, Valeria Tessarollo, Ilaria Costantino, TransiDEA Group, Maurizio Bonati

**Affiliations:** 1 Laboratory of Child Health and Development Epidemiology, Department of Medical Epidemiology, Istituto di Ricerche Farmacologiche Mario Negri IRCCS, Milan, Italy; 2 Department of Health Sciences, University of Milan, Milan, Italy; 3 Department of Translational Medical Sciences, Child and Adolescent Neuropsychiatry, University of Naples Federico II, Naples, Italy; 4 Department of Maternal and Child Health, UOSD of Child and Adolescent Psychiatry, AOU Federico II, Napoli, Italy; 5 UOC Neuropsichiatria Infantile Ospedale di Macerata, Macerata, Italy; 6 Centro Psicodiagnostico Italiano, Milan, Italy; 7 Information Science for Clinical Knowledge Sharing Unit, Department of Medical Epidemiology, Istituto di Ricerche Farmacologiche Mario Negri IRCCS, Milan, Italy; 8 Child Neuropsychiatry Unit, Department of Health Sciences, ASST Santi Paolo e Carlo, San Paolo Hospital, Università degli Studi di Milano, Milan, Italy

**Keywords:** ADHD, AMHS, CAMHS, transition care

## Abstract

**Aims:**

Ensuring a successful transition to Adult Mental Health Services (AMHS) is fundamental for attention deficit/hyperactivity disorder (ADHD) patients to prevent adverse scenarios in adults (e.g., psychiatric disorders, substance or alcohol abuse). Yet, most European nations do not have appropriate transition guidelines and still fail to adequately support transition processes. This study aims to enquire about the current transition paths in Italy and the perceived experiences of the patients and their clinicians.

**Methods:**

The present observational study collected 36 interviews with young adults with ADHD who turned 18 between 2017 and 2021. Simultaneously, two questionnaires were filled in by the clinicians (both from paediatric and AMHS) who were involved in their transition paths. These tools collected information about the transition process, the services that cared for the young adults and well-being indicators such as impairment in daily life, employment status and the presence of sentinel events (e.g., critical stage accesses to the emergency room or hospitalizations). Successful and failed referrals were analysed.

**Results:**

A referral to an AMHS was attempted for 16 young adults (8 before age 18 and 8 when turning 18), and 8 patients (22.2% overall) were successfully taken into the care of the AMHS. Twenty patients were not referred since it was deemed unnecessary (*N* = 6) or because of the lack of specialized services or compliance (*N* = 14). At the time of the interview, only nine participants were still under AMHS care. Of eleven individuals with a high need for care (identified by the level of impairment, support needs or sentinel events), five were not followed by a mental health professional at the time of the interview.

**Conclusions:**

For the majority of ADHD young adults, a transition path was never started or completed. While this is partly due to mild levels of impairment, in many cases it was difficult to find a service that could care for the adult patient. Only one out of four young adults are successfully transferred to AMHS care. Creating or improving evidence-based transition guidelines should be a priority of the public health system to ensure healthcare for as many patients as possible. The results of this study will converge towards the need for recommendations for the transition of services from adolescence to adulthood for young people with ADHD for Italian clinical practice.

## Introduction

Attention deficit/hyperactivity disorder (ADHD) is a neurodevelopmental disorder with a prevalence of around 5% in childhood and adolescence (Roughan and Stafford, 2019; Seixas *et al.*, 2012). About 7% of the patients continue to have impairing symptoms in adulthood (Song *et al.*, 2021). Moreover, ADHD in adults has a high comorbidity with psychiatric disorders, substance or alcohol abuse, antisocial personality or even criminal activities (Di Lorenzo *et al.*, 2021). To prevent the onset of adverse scenarios, it is fundamental not to lose patients when they stop being cared for by Child and Adolescent Mental Health Services (CAMHS). Instead, too often, the apparent lack of severe symptoms hinders a successful transition to Adult Mental Health Services (AMHS) (McCarthy *et al.*, 2009; Young *et al.*, 2011).

Several factors contribute to this discontinuity of care in adolescence: lack of organization, scarce resources and collaboration between services, stigma, concerns about peers judgement and low family compliance (Anderson *et al.*, 2022; Roberti *et al.*, 2023a). The result is feeling left alone (Driver *et al.*, 2022) and a later worsening of symptoms with urgent service re-entries (Anderson *et al.*, 2022).

While some guidelines exist at an international level (e.g., the National Institute for Clinical Health and Excellence [NICE] Transition Guidelines and the Ready Steady Go program in the U.K., The Six Core Elements in the U.S.A.) (Meyers and Irwin, 2023; National Institute for Health & Care Excellence, 2016; White *et al.*, 2018), their feasibility at a practical level is questioned (Eke *et al.*, 2020, 2019; Signorini *et al.*, 2017). Moreover, not all countries possess such regulations, as is the case for most European nations. The improvement of transition guidelines should be a priority of the healthcare system. This need is particularly evident and urgent in Italy: in a country where the estimated ADHD prevalence ranges from 1.1 to 3.1% among youth aged 5–17 years (Reale and Bonati, 2018), no shared transition practices by CAMHS and AMHS currently exist. This observation emerged within the first phase of the *Transition care between adolescent and adult services for young people with chronic health needs in Italy* project (TransiDEA – Transition in Diabetes, Epilepsy and ADHD patients), designed to assess the current state of transitioning practices, the experience of patients, their families and the clinicians of both CAMHS and AMHS (Roberti *et al.*, 2023a). Indeed, amongst 42 services that participated in the 2022 Survey (Phase 1), only 21 declared having a transition protocol, and even fewer (6 in total) provided a copy. A fragmented picture emerged from the analysis of such protocols (Roberti *et al.*, 2023b). The goal of the present study (Phase 2) was to describe the transition pathways in terms of referral to AMHS outcome, continuity of care, well-being indicators and perceived experiences of the patients and their CAMHS and AMHS (in case of a successful transition) clinicians in three Italian services. Putting together the information collected in phases 1 and 2 will lead to the definition of a consensus document to be adopted in Italian services (Phase 3 of the TransiDEA project, not reported in this paper).

## Methods

**Figure 1.**
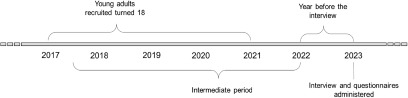
Timeline of the patients’ path. The participants of the present study turned eighteen between 2017 and 2021. They were all interviewed in 2023, and questions were related to the “last year”. Other questions referred to the time between young adults turning 18 and the year before the interview, defined as the “intermediate period.”

The present observational study was conducted between January and July 2023 as a part of the TransiDEA project – ADHD branch. The goal was to collect at least 30 semi-structured interviews (10 from the north, 10 from the centre and 10 from the south of Italy) of young people who turned 18 within the past 3–6 years (approximately 2017–2021). The interviews aimed to collect information about patients when they turned 18, in the year before the interview, and in the intermediate period (i.e., the time between turning 18 and the year preceding the interview) (Fig. 1). In parallel, two questionnaires were developed for the CAMHS and AMHS clinicians involved in the transition paths. These tools were designed based on Phase 1’s results and discussed with a small group of clinicians, and adjustments based on their feedback were made. Specifically, the content of the interview enquired about:

- ADHD type, comorbidities treatments and impairment areas in daily life;
- Services that cared for the young patient before turning 18, in the year before the interview and in the intermediate period;
- The severity of symptoms, treatments and occupation at the time of the interview, as well as the presence of sentinel events (critical stage accesses to the emergency room or hospitalizations due to, for example, accidents, fights, self-harm, etc.);
- Organization of the transition process (ages of transition planning and actual passage, information sharing, family involvement, activities such as appointments and spaces to express doubts and needs);
- In case of failed passage, the reason and the later need for care from public or private healthcare professionals;
- Involvement of general practitioner (GP);
- Perception of self as an adult ADHD patient, and advice that they would give to younger people about to approach transitioning age.

The questionnaires replicated questions on ADHD symptoms, their severity and impairment areas in daily life, as well as on the organization of the transition process. Moreover, we asked whether they asked for feedback (CAMHS) or sent feedback (AMHS) on the transition’s success. The clinicians filled in the questionnaires independently, while the interviews were administered by a CAMHS worker who did not follow the patients during their care path. The questionnaires and the interview are available in the supplementary material.

Since questions included in points (a), (b), (c), (f) and (g) of the interviews were asked to all participants, while those in points (d) and (e), respectively, only to referred and non-referred patients, analyses will be reported in separate blocks. The first part will describe the whole sample (e.g., clinical characteristics, occupation, daily life functioning), and the second will focus on details of non-referred and referred patients. The analyses reported are descriptive. Data are reported as the number and percentage of responders and tested using the chi-square exact test, where applicable. Median values and standard deviations summarize continuous variables.

The centres involved were ASST Santi Paolo e Carlo – San Paolo Hospital, Milan, Federico II University Hospital, Naples, and Macerata Hospital, Macerata. The ethics committee of the coordinating centre, the IRCCS ‘Carlo Besta’ Foundation Ethics Committee, approved the study (9 November 2022, protocol n. 09). All participating centres notified their ethics committees. The study followed the ethical standards of the 1964 Declaration of Helsinki and its later amendments. We followed the Strengthening the Reporting of Observational Studies in Epidemiology (STROBE) reporting guidelines.

## Results

### Descriptive characteristics of the sample

**Figure 2.**
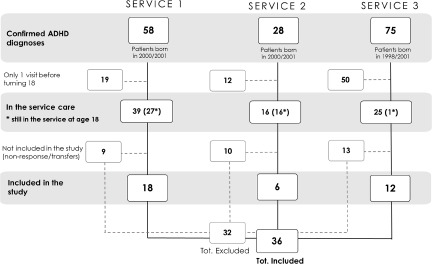
Flowchart representing the number of ADHD patients born in the target years for each service involved in the study and their continuation of care path with the service up until the study.

Thirty-six interviews (18 from the north, 12 from the centre and 6 from the south) were conducted, in 13 cases with the participants’ parents (8 mothers and 5 fathers). The median age of the young adults at the time of the interviews was 22 years (*SD* = 1.3). According to ADHD gender prevalence (Bonati *et al.*, 2021; Cortese *et al.*, 2023), 34 males and 2 females participated. The flowchart (Fig. 2) reports the number of patients involved in the study for the three services and the different care paths before, during and after transition. Upon reaching adulthood, the rate of patients in care at the paediatric service differed widely between participating services (from 4 to 69%).

**At the diagnosis time**, most patients (*N* = 35) had combined ADHD (i.e., with both inattentive and hyperactive-impulsive symptoms), while only one had ADHD with predominant inattention. Two-thirds of the patients received an ADHD diagnosis during primary school, while the remaining (36%) were diagnosed later. Almost all diagnoses occurred within the public healthcare system (94%), with only two cases of diagnoses in the private sector. Twenty-three patients (64%) also had one or more comorbidities: learning disorders affected 39% of patients, oppositional-provocative/behaviour disorder 25%, mood disorders 8% and autism spectrum disorder 6%. Intellectual disability, sleep disorder and mixed specific developmental disorders were diagnosed in 3% of ADHD patients. Level of impairment **at transition** was mild in 15 cases (42%), moderate in 14 (39%) and medium/severe in 7 (19%). When turning 18, the areas of impairment most frequently reported by the CAMHS clinicians were: education/work (35 patients), emotionality (14), social/emotional relationships (13), impulsivity (11) and general quality of life (6).

Employment status **at the time of the interview** was also assessed. Seventeen young adults (47%) were working, eleven (31%) were still in high school or college and eight were not studying and were not employed. No significant differences emerged when comparing employment between participants with mild and moderate, medium or severe impairment (Chi-square = 0.18, *p* > 0.99). In particular, of the 15 with mild impairment, only 3 (20%) were not employed; 3 out of 13 (23%) with moderate and 1 out of 7 (14%) with medium or severe impairment were unemployed. Twenty-one participants (58%) were completely independent in daily life. The remaining 42% declared needing support with practical-organizational skills (e.g., manual dexterity, fine-motor skills, studying/ working, planning, economic and bureaucratic management, and technology use). CAMHS clinicians reported that the median duration of CAMHS stay was 4 years (*SD* = 2.7 years; range, 1–10 years), mostly (69%) since diagnosis.

**Figure 3.**
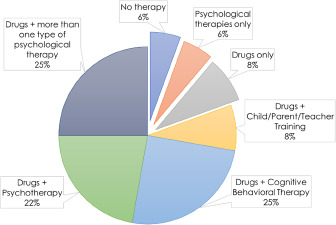
Types of treatment reported by the thirty-six young adults interviewed.

Regarding **treatment**, all participants except for two received pharmacological or psychological therapy in CAMHS. Most patients (81%) received combined therapy: pharmacological and psychological (cognitive behavioural therapy, child/parent/teacher training or psychotherapy). Some also combined different types of psychological treatment (Fig. 3). Two participants underwent only psychological therapy, while three only pharmacological treatment. Methylphenidate was the medication most commonly prescribed for ADHD; 31 young adults (86% of the entire sample) reported taking it. Of these, 24 (77%) stopped it, while 7 still took it when they turned 18. Atomoxetine was prescribed in the past to five patients (14% of the sample) and then stopped for all of them.

All participants were also asked about the GP’s role. Only six young adults reported that their GPs had been involved in their ADHD care path. Five said they did not need them as they preferred communicating with the specialists, but two also reported that they felt a lack of expertise for their condition. Nine did not feel the need to involve the GPs.

### Referred vs. non-referred patients

For **7 out of 36** patients, **a referral was not considered necessary** by CAMHS clinicians (one continued accessing CAMHS for medication prescriptions).

Of the remaining 29 patients for whom further care was deemed necessary, a **referral to a public AMHS was not carried out for 13 patients**. For nine of them, it was difficult to find an AMHS, and in time they were lost. Three patients dropped out of care, whereas one young adult opted for a private service. At the interview, eleven of these thirteen patients were still not in care (two attempted private care, but then stopped it), one tried to be taken into the care of an AMHS and then chose private care, and one decided autonomously to attend an AMHS due to drug addiction (and is still cared for by that service).

A **referral** to an AMHS was attempted for **16** young adults. For eight of them, the referral was made **before the age of 18**, and for the other eight, when turning 18.

Access to AMHS was successful in only 50% of the cases. Eight patients did not undergo a complete transition path: one was lost and then returned spontaneously to AMHS, two chose a private care path, one attempted private care but is currently not in care, one was not referred to an AMHS but transferred to a territorial CAMHS and has not changed service since, and three never attended any service again. Among the eight patients whose referral was successful, six continued their treatment at the AMHS without significant interruptions (i.e., the only six patients of the sample for which continuity of care was guaranteed), one left AMHS for an extended period and then contacted a service again, and one dropped out.

**Figure 4.**
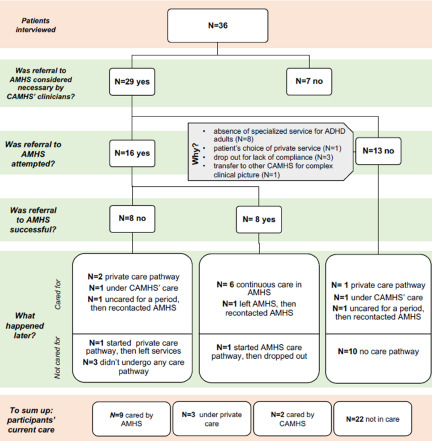
Paths of referred vs. non-referred patients.

Figure 4 summarizes the paths of referred vs. non-referred patients. A complete summary of services that cared for the patients before turning 18, in the subsequent years, and at the time of the interview, as well as some indicators of the clinical picture, is provided in the supplementary material.

Amongst the referred patients (*N* = 16), the referral was made through informational meetings between CAMHS workers and the patient and their family in three cases. Only in one case was a case discussion between paediatric and adult services organized. For four patients, information regarding the clinical history was exchanged by a diagnostic report. The patients and their families reported significant variability between referral and assessment at the adult service: 10 months (median, 10.5; SD, 10.4), with a minimum of 1 month to a maximum of 36 months. Clinicians’ responses also reflected this picture, indicating a waiting time of 7 months (median, 8; SD, 8), with a minimum of 1 month to a maximum of 24 months. Five CAMHS clinicians stated that more time would be needed, indicatively an additional 6 months.

In only one case, the CAMHS providers asked for feedback on the patient’s admission in AMHS. No follow-up meetings were organized between the services. Four of thirty-one patients continued taking methylphenidate, one was taking risperidone, and one methylphenidate and risperidone, while two were not taking any medication.

To summarize, at the interview, **27** patients were **not cared for by a public AMHS**. They were either unsupervised (22 patients), privately cared for (3) or only afferent to CAMHS for prescription medication (2). Therefore, **nine participants** were **in AMHS care at the time of the interview** (two were also cared for by private professionals). For these nine patients, a possible referral was first addressed when they were 17 (median) and completed after they turned 18 (median; SD, 0.93). Five of the **nine participants in AMHS care at the time of the interview** had moderate, two medium/severe and two mild levels of impairment. Only one reported sentinel events in the year preceding the interview due to substance abuse.

### Well-being indicators

At the interview, two out of seven adolescents with a medium/severe **impairment** were cared for by an AMHS, while three were cared for by a private mental health professional, and two dropped out. Eight out of fourteen adolescents with a moderate impairment were not cared for by a healthcare service (four of whom required support for daily life activities and/or presented sentinel events), five attended an AMHS and one remained in a CAMHS.

Eight patients presented at least one **sentinel event**, and six were never referred (even if their clinicians evaluated half of them as needing further care). Of the 27 young adults not currently cared for by an AMHS (17 with good general well-being, 10 with marked problems in various areas of life), in the year preceding the interview, 1 case of emergency room admission following fights, 1 follow-up examination after a drunk driving episode, 1 substance abuse and 4 episodes of car accidents were reported.

Nineteen patients reported a need for support in daily life activities and/or a sentinel event in the year before the interview: eight sought a mental health professional (six AMHS, two private), while eleven were unsupervised. **Eleven individuals with a high need for care** could be identified based on the degree of impairment (moderate or medium/severe) associated with support needs or sentinel events. Five of these were not followed by a mental health professional at the time of the interview. Moreover, three were among those patients for whom a referral was deemed unnecessary.

When comparing the above indicators, it can be observed that the level of impairment and the proportion of young adults needing support for daily life activities is higher in patients attending an AMHS and in those with an unsuccessful referral. On the contrary, the rate of sentinel events is greater in adults not referred to an AMHS (Table 1). The proportions of the indicators is similar in those with a successful versus unsuccessful referral.

**Table 1.** Number of patients presenting level of impairment moderate or medium/severe, need for support, presence of sentinel events and unemployment. These patients are grouped into those for which a referral was successful, unsuccessful or not carried out (respectively, 9, 7 and 20 in the full sample)

	AMHS (*N* = 9)	Unsuccessful (*N* = 7)	Not referred (*N* = 20)
Moderate or medium/severe impairment	7	5	9
Need for support (yes)	5	3	7
Sentinel events (yes)	1	1	6
Unemployment	5	1	2

### Acquired skills, specific experiences

All 36 young adults were asked questions to explore their perception of skills acquisition during the transition. Almost all (eight out of nine) of the patients currently in AMHS care reported not having acquired any particular skills. In contrast, a similar perception for those not in care was present for only 37% (10 participants). As for the description of their transition experience, unassisted young adults and those under AMHS care reported very similar observations. For instance, they found it difficult to handle everyday life, especially in organizational aspects and social exchanges, with difficulty managing anger and impulsiveness. They agreed on the importance of getting help from close people and trusting doctors. While they also agreed that in time, it became easier to learn how to manage symptoms, only young adults still in AMHS care stressed the importance of taking medications and preparing for the passage to adult services. On the other hand, the unassisted young adults reported greater difficulties in the school/work environment and marked as desirable more practical strategies and support from teachers. Some of the comments made by the young adults during the interviews are presented in Box 1.

**Table:** 

**Box 1.** Some of the comments made by young adults to the questions ‘In your opinion, what was missed or needed to be done differently during your journey?’ and ‘What advice would you give to younger people with ADHD still being cared for by CAMHS?’. The first column shows the comments of referred patients and the second column those of non-referred patients.	
**Referred young adults**	**Non-referred young adults**
***In your opinion, what was missed or needed to be done differently during your journey?***	
*All went well, but I had some concerns about the transfer to the territorial adult facility, as it has few resources.*	*Nothing in particular was missing; I think what was needed was done.*
*All went well in the CAMHS, but I found more difficulties in the pathway of the transition to adult facilities*	*I would have needed more follow-up after the age of 18. I had a depressive period that I think was related to the ‘clash’ with adult reality after school ended …. Preparing kids for what will happen in their lives is essential.*
*More informative/educational pathway starting at an early age.*	*When I was being cared for, I was not very aware of my situation. Maybe it would have been helpful to continue the visits and treatments even after I reached a state of awareness, so more ‘grown up’, perhaps I would have used them more. Now, for example, I am also becoming more and more aware because my girlfriend is going through an autism spectrum diagnosis, and this has aroused a lot of curiosity in me, so I am getting more and more information.*
*I don’t know; as a child, I was not aware enough to understand it, but I think the therapy was adequate. Certainly, it would have been helpful to have more continuity afterward as well.*	*I had a lot of difficulties in high school. I could not admit that I had a disorder, and I was ashamed. The environment was not very tolerant, and I feared being made fun of. It would be essential to raise awareness in school about these issues (ADHD, learning disorders, etc.); it would take very little to inform people and make them accept each other. They always have meetings on sex education, drugs, etc., I don’t see why it can’t be done on these issues as well; it would be so important.*
*It was important to continue a care path even as an adult.*	*In my teenage years, I had thought that my parents had been wrong to give me support because it bothered me to be diverse. Now, putting myself in their shoes, I would have done the same thing.*
*I would change nothing. Overall, it went well.*	
***What advice would you give to younger people with ADHD still being cared for by CAMHS?***	
*To ask questions from an early age about how they work and not to be afraid to take the medications.*	*Start cognitive behavioural psychotherapy immediately to gain awareness and promote appropriate symptom management.*
*Learn to manage symptoms because they also impact future performance at work and in life in general. Turn to private care because you will be more supported.*	*It is a condition that you can live with and still have very good results.*
*I would explain what they are up against and say always to follow what the clinicians recommend without doing ‘your own thing’. It could be a tough change. In any case, I realized that you have to trust the clinicians.*	*That the disorder never goes away, even if the characteristics change. In any case, having a normal everyday life is still possible. More practical strategies to become more independent, especially at the school level, would have been helpful.*
*It depends from person to person. For me, it went quite well, I didn’t feel ‘abandoned’ even when changing services. It’s still a different approach, you get less ‘pampered’. I would say to listen as much as possible to the advice of those who follow and help us.*	*I don’t know, I’ve never been particularly preoccupied with thinking about ADHD. I would have needed more support in school and in studying. I had the support teacher, but I still struggled a lot.*
*I would say that ADHD is not necessarily bad; there are positive aspects, such as the intense interest one achieves in some specific areas. In any case, it is all very tiring, and it always seems that others around are ‘better’. I would advise not to neglect one’s hyperactivity and always try to relieve stress in a healthy way, without forcing oneself to sit still for a long time.*	*I would say that they should not feel blocked by the situation at the time because it may also improve spontaneously over time. I remember that the process needed to arrive at the diagnosis was complicated, mainly because the teachers did not recognize my difficulty. I remember so many ‘walls’ before arriving at defining a path.*
*I don’t know; it’s hard to answer that question. It’s a personal aspect, and I know that not everyone is like me. It’s important to learn how to relate to others. Then I realized that you have to be very careful about ‘developmental risk’ – we ADHD kids are particularly at risk with drugs because we physiologically need to feel certain strong emotions … So if we are not well aware and prepared, it’s a snap to ‘get into it’, just try it even once, and it’s too late. I realized this to my own expense, unfortunately.*	*I would say to use the opportunities to learn more about your problem. I wouldn’t know what to say about treatments because, as a kid, I used to take them a little bit lightly, I never took much interest. My friends helped me greatly because I always felt understood and welcomed.*
*It’s ‘quite a mess’ because it is never clear how to behave, what to do, etc. It is important not to disengage from services after age 18; I would advise against making the same mistake I made. As adults, we feel like ‘heroes’ who can do and decide everything on our own, but it is very easy to end up in bad situations.*	*I would say that the more they grow up, the more the world will be in their hands. I used to live in a ‘parallel world’ and was not responsible for myself. I would also say to be very careful handling interpersonal relationships: people do not necessarily understand and accept how we are, and you must be strong.*
	*I wouldn’t wish anyone to feel as bad as I did (I went through a bad depressive period when I was 18). Don’t preclude yourself from anything, and don’t be limited by what others say. For example, I was advised to do a 3-year professional school, but I insisted and completed a traditional 5-year school course.*
	*I would say that it is important to be aware that you are a little different from others, to live with it, and accept it as much as possible. If you fixate on being ‘sick’, you are only missing out on great possibilities …. It is better to try to adapt to the demands ‘of the world’ to work and fit in than to be rigid and expect others to accept us completely. Being totally ‘yourself’ without making any effort and being excluded from society is much worse. One must try to turn difficulties into something ‘fun’, know how to manage oneself in social contexts, and learn to adapt as much as possible. It is difficult in today’s world, at least among adults, to declare that you have this disorder because society is not yet ready. You will always be one less good, quick-witted, and attentive at work than others.*
	*I would say to be very careful, and if symptoms worsen at any moment, seek help immediately.*

## Discussion

Clinics dedicated to transition were developed in France (Le Roux *et al.*, 2023), whereas elsewhere, programs such as those of the Got Transition Six Core Elements in the United States, the NICE Transition Guidelines and Ready Steady Go in the United Kingdom (van Staa *et al.*, 2020) were set up. However, even if research and innovative practices in transitional care for young people with chronic conditions have improved in the last few years, barriers remain. Many people experience discontinuity of care and drop out during this critical period (Reneses *et al.*, 2023). Inadequate transition processes not only pose risks to mental health, but can also exacerbate the likelihood of engaging in at-risk behaviours. They may even contribute to inequalities in care, which every individual should be guaranteed as a fundamental human right (Munyikwa *et al.*, 2023). Supporting patients with ADHD during transitions is crucial, and clinicians must be empowered to facilitate an appropriate transition process (Scarpellini and Bonati, 2022).

The results here reported indicate the urgency of timely interventions to facilitate the transition of ADHD patients from paediatric to adult services. This successful transition rate is similar to that described in other countries (Eke *et al.*, 2020; Maurice *et al.*, 2022), but still inadequate. Low referral acceptance and lack of communication between CAMHS and AMHS contribute to the loss of care for approximately half of patients. Such mechanisms result from scarce services and resources, as previously highlighted elsewhere (Appleton *et al.*, 2023; Eke *et al.*, 2020; Roberti *et al.*, 2023a).

The first result of the present study is that a referral to an AMHS was carried out in nearly half of the cases. Often, patients were not referred since the CAMHS clinicians evaluated the level of impairment as mild. Nonetheless, for a non-negligible number of cases, it was challenging to find a service that could care for adult patients. The situation is even worse when considering that only one out of four young patients deemed in need of continued treatment by psychiatrists were successfully transferred to AMHS. Moreover, it is worrying that only one out of three patients with medium/severe impairment and nearly half of the subjects with a potentially high level of care needs attended AMHS at the time of the interview.

The second main finding is that transition paths are related to the type of child psychiatric service (Fig. 2). While the most common scenario in a university hospital (service 2) is an early referral of patients, most patients are referred at 18 years of age in a territorial structure associated with a university (service 1). On the other hand, in service 3, continuity of care appears to be difficult as patients are lost even before turning 18. Nonetheless, 33% of the patients were described as still having a medium impairment level, and for 25% of them, a referral to adult services would have been necessary. This difference between services may be due to each hospital’s different networks and resources. In the future, care policies should address these differences. These three services had previously been asked whether they had a protocol to guide transition processes. (Roberti *et al.*, 2023a) Only service 1 and service 2 responded positively. Although from the analysis of such documents we observed several limitations (Roberti *et al.*, 2023b), the mere existence of a formal transition document might contribute to the continuity of care for patients in services 1 and 2.

When viewing the transition pathways, a few patients had already been transferred to AMHS when they turned 18, while more were transferred in the following years. Notably, only nine were still in care at the interview. The proportion of referred patients and successful referrals is the same when restricting the analysis to the homogeneous sample of patients who were still in the CAMHS at 18 years of age, suggesting that our findings are scantly influenced by the dropout of the adolescents from CAMHS before 18 years of age.

The dropout from the AMHS is an inconvenience of the treatment path that must be carefully evaluated considering the possible outcomes. In fact, of the nine patients cared for by an AMHS, six had a continuous care path, while three were lost in the intermediate period and then returned following a worsening of symptoms. Two of them reported having developed drug addiction in the meantime. They described their experience as follows: ‘… We ADHD kids are at risk with drugs because we physiologically need to feel strong emotions. If we’re not well aware and prepared, it’s a snap to “get into it”, try it even once, and it’s too late. I realized this to my own expense, unfortunately’, and ‘It is important not to disengage from services after age 18; I would advise against making the same mistake I made. As adults, we feel like “heroes” who can do and decide everything on our own, but it is very easy to end up in bad situations’.

Other sentinel events were highlighted in the study, i.e., critical episodes that lead to emergency room access in the healthcare system, recurrent relational difficulties, reactivity and impulsivity, attentional difficulties, anxious symptomatology, as well as being unemployed. Unemployment also seemed common among those who were not referred to an AMHS, but would have needed it. As already described, the risk of unemployment in ADHD young adults is high, even up to 70% (Helgesson *et al.*, 2023). Episodes of fights, drunk driving episodes and car accidents must not go unnoticed. In our sample, these episodes were reported in 88% of the cases by these patients who were not in AMHS care. It follows that it is essential that warning signs are promptly ascertained and reported whenever they appear. Therefore, GPs (who seem not currently involved in ADHD management) should be encouraged to advise their patients to resume a specific care path should they detect these signs. The GP’s role can be particularly relevant when considering that half of the patients with a high need for care were not cared for by a mental health professional.

Lastly, considering that universal healthcare is guaranteed in Italy, it is striking that one in five patients felt the need to contact a private mental health professional at least once during the care pathway, particularly after leaving the CAMHS.

It should be kept in mind that the description provided by the present study is limited to a few Italian centres and that the sample was recruited voluntarily and could be biased. Consequently, the numbers reported are small and cannot be representative of the transition pathways management of all services. We, however, managed to represent a variety of situations from independence in daily life (present for 58% of young adults) to drugs and therapies administered, and several types of paths and clinical case complexities, i.e., the real world. Yet, this study has the merit to be the first one to provide a complete description of transition pathways in different Italian geographical areas, from both young adults and their families and the clinicians’ perspectives. The emerging figure is worrying, although not exclusive to Italy, and should encourage us to promptly undertake improvement initiatives (Leavey *et al.*, 2019; Swift *et al.*, 2013). It is crucial to further explore the factors that lead to unsuccessful referrals in the future. Other than the lack of resources, dedicated specialized public services and follow-up, the transition period per se should also be analysed carefully. The TransiDEA project’s third phase will put together the information collected in phases 1 and 2, and aim to design a process that begins earlier and is participatory among the different providers involved, the young adults and their families. This will allow us to jointly define a consensus document to be adopted in Italian services, to inform a more appropriate transition pathway by involving clinicians, young adults, scientific societies and patient associations.

While the defined consensus document will be drafted based on the experiences of young adults and services in Italy, we hope that it will encourage other nations to replicate a similar model.

## Supporting information

Roberti et al. supplementary material 1 Roberti et al. supplementary material

Roberti et al. supplementary material 2 Roberti et al. supplementary material

## Data Availability

The data presented in this study are available on the Zenodo platform (https://zenodo.org/doi/10.5281/zenodo.10843581). The materials used (structured interview and questionnaires) are available online as supplementary material.

## Supplementary material

The supplementary material for this article can be found at https://doi.org/10.1017/S2045796024000544.

## References

[ref1] Anderson JK, Newlove‐Delgado T and Ford TJ (2022) Annual Research Review: A systematic review of mental health services for emerging adults – Moulding a precipice into a smooth passage. *Journal of Child Psychology and Psychiatry* 63, 447–462. 34939668 10.1111/jcpp.13561

[ref2] Appleton R, Canaway A, Tuomainen H, Dieleman G, Gerritsen S, Overbeek M, Maras A, van Bodegom L, Franić T, de Girolamo G, Madan J, McNicholas F, Purper-Ouakil D, Schulze UME, Tremmery S and Singh SP (2023) Predictors of transitioning to adult mental health services and associated costs: A cross-country comparison. *BMJ Mental Health* 26, e300814. 10.1136/bmjment-2023-300814 PMC10603405 37879676

[ref3] Bonati M, Scarpellini F, Cartabia M, Zanetti M and on behalf of the Lombardy ADHD Group (2021) Ten years (2011–2021) of the Italian Lombardy ADHD Register for the diagnosis and treatment of children and adolescents with ADHD. *Children* 8, 598. 10.3390/children8070598 PMC8304222 34356577

[ref4] Cortese S, Song M, Farhat LC, Yon DK, Lee SW, Kim MS, Park S, Oh JW, Lee S, Cheon K-A, Smith L, Gosling CJ, Polanczyk GV, Larsson H, Rohde LA, Faraone SV, Koyanagi A, Dragioti E, Radua J, Carvalho AF, Il Shin J and Solmi M (2023) Incidence, prevalence, and global burden of ADHD from 1990 to 2019 across 204 countries: Data, with critical re-analysis, from the Global Burden of Disease study. *Molecular Psychiatry* 28, 4823–4830. 37684322 10.1038/s41380-023-02228-3

[ref5] Di Lorenzo R, Balducci J, Poppi C, Arcolin E, Cutino A, Ferri P, D’Amico R and Filippini T (2021) Children and adolescents with ADHD followed up to adulthood: A systematic review of long-term outcomes. *Acta Neuropsychiatrica* 33, 283–298. 34384511 10.1017/neu.2021.23

[ref6] Driver D, Berlacher M, Harder S, Oakman N, Warsi M and Chu ES (2022) The inpatient experience of emerging adults: Transitioning from pediatric to adult care. *Journal of Patient Experience* 9, 237437352211336. 10.1177/23743735221133652 PMC9597024 36311907

[ref7] Eke H, Ford T, Newlove-Delgado T, Price A, Young S, Ani C, Sayal K, Lynn RM, Paul M and Janssens A (2020) Transition between child and adult services for young people with attention-deficit hyperactivity disorder (ADHD): Findings from a British national surveillance study. *The British Journal of Psychiatry* 217, 616–622. 31159893 10.1192/bjp.2019.131 PMC7589988

[ref8] Eke H, Janssens A and Ford T (2019) Review: Transition from children’s to adult services: A review of guidelines and protocols for young people with attention deficit hyperactivity disorder in England. *Child and Adolescent Mental Health* 24, 123–132. 32677178 10.1111/camh.12301

[ref9] Helgesson M, Björkenstam E, Rahman S, Gustafsson K, Taipale H, Tanskanen A, Ekselius L and Mittendorfer-Rutz E (2023) Labour market marginalisation in young adults diagnosed with attention-deficit hyperactivity disorder (ADHD): A population-based longitudinal cohort study in Sweden. *Psychological Medicine* 53, 1224–1232. 35275515 10.1017/S0033291721002701 PMC10009402

[ref10] Leavey G, McGrellis S, Forbes T, Thampi A, Davidson G, Rosato M, Bunting B, Divin N, Hughes L, Toal A, Paul M and Singh SP (2019) Improving mental health pathways and care for adolescents in transition to adult services (IMPACT): A retrospective case note review of social and clinical determinants of transition. *Social Psychiatry & Psychiatric Epidemiology* 54, 955–963. 30843086 10.1007/s00127-019-01684-z

[ref11] Le Roux E, Bourmaud A, Jacquin P, Mahlaoui N, Guffroy A, Belot A, Romier M, Sattoe J, Van Staa A, Alberti C, Mellerio H and Dumas A (2023) Clinics dedicated to transition preparation for adolescents and young adults with chronic conditions: Factors influencing their use. *Archives de Pédiatrie* 30, 617–619. 37704524 10.1016/j.arcped.2023.08.004

[ref12] Maurice V, Russet F, Scocco P, McNicholas F, Santosh P, Singh SP, Street C and Purper-Ouakil D (2022) Transition from child and adolescent mental health care to adult services for young people with Attention-Deficit/Hyperactivity Disorder (ADHD) or Autism Spectrum Disorder (ASD) in Europe: Barriers and recommendations. *L’Encéphale* 48, 555–559. 10.1016/j.encep.2022.01.012 35725512

[ref13] McCarthy S, Asherson P, Coghill D, Hollis C, Murray M, Potts L, Sayal K, de Soysa R, Taylor E, Williams T and Wong ICK (2009) Attention-deficit hyperactivity disorder: Treatment discontinuation in adolescents and young adults. *British Journal of Psychiatry* 194, 273–277. 10.1192/bjp.bp.107.045245 19252159

[ref14] Meyers MJ and Irwin CE (2023) Health care transitions for adolescents. *Pediatrics* 151, e2022057267L. 10.1542/peds.2022-057267L 37010403

[ref15] Munyikwa M, Hammond CK, Langmaid L and Ratner L (2023) Growing up can be hard to do: Reimagining 1 structurally supportive pediatric-to-adult transitions of care from a rights-based perspective. *Health and Human Rights* 25, 51–65. 37266310 PMC9973513

[ref16] National Institute for Health & Care Excellence (2016) NICE Clinical Guideline [NG43] Transition from children’s to adults’ services for young people using health or social care services.

[ref17] Reale L and Bonati M (2018) ADHD prevalence estimates in Italian children and adolescents: A methodological issue. *Italian Journal of Pediatrics* 44, 108. 10.1186/s13052-018-0545-2 PMC6126024 30185215

[ref18] Reneses B, Escudero A, Tur N, Agüera-Ortiz L, Moreno DM, Saiz-Ruiz J, Rey-Bruguera M, Pando M-F, Bravo-Ortiz M-F, Moreno A, Rey-Mejías Á and Singh SP (2023) The black hole of the transition process: Dropout of care before transition age in adolescents. *European Child and Adolescent Psychiatry* 32, 1285–1295. 35048161 10.1007/s00787-021-01939-8 PMC10276128

[ref19] Roberti E, Scarpellini F, Campi R, Giardino M, Clavenna A and Bonati M (2023a) Transitioning to adult mental health services for young people with ADHD: An Italian-based survey on practices for pediatric and adult services. Preprint, In Review 10.1186/s13034-023-00678-9 PMC10685479 38017552

[ref20] Roberti E, Scarpellini F, Campi R, Giardino M, Clavenna A and Bonati M (2023b) Protocols for transitioning to adult mental health services for adolescents with ADHD. Preprint, Public Health and Healthcare. 10.1186/s12888-024-06011-8 PMC11378623 39237898

[ref21] Roughan LA and Stafford J (2019) Demand and capacity in an ADHD team: Reducing the wait times for an ADHD assessment to 12 weeks. *BMJ Open Quality* 8, e000653. 10.1136/bmjoq-2019-000653 PMC6830462 31750403

[ref22] Scarpellini F and Bonati M (2022) Transition care for adolescents and young adults with attention‐deficit hyperactivity disorder (ADHD): A descriptive summary of qualitative evidence. *Child: Care, Health and Development*, cch.13070. 10.1111/cch.13070 36223008

[ref23] Seixas M, Weiss M and Müller U (2012) Systematic review of national and international guidelines on attention-deficit hyperactivity disorder. *Journal of Psychopharmacology* 26, 753–765. 21948938 10.1177/0269881111412095

[ref24] Signorini G, Singh SP, Boricevic-Marsanic V, Dieleman G, Dodig-Ćurković K, Franic T, Gerritsen SE, Griffin J, Maras A, McNicholas F, O’Hara L, Purper-Ouakil D, Paul M, Santosh P, Schulze U, Street C, Tremmery S, Tuomainen H, Verhulst F, Warwick J and De Girolamo G (2017) Architecture and functioning of child and adolescent mental health services: A 28-country survey in Europe. *The Lancet Psychiatry* 4, 715–724. 28596067 10.1016/S2215-0366(17)30127-X

[ref25] Song P, Zha M, Yang Q, Zhang Y, Li X and Rudan I (2021) The prevalence of adult attention-deficit hyperactivity disorder: A global systematic review and meta-analysis. *Journal of Global Health* 11, 04009. 10.7189/jogh.11.04009 PMC7916320 33692893

[ref26] Swift KD, Hall CL, Marimuttu V, Redstone L, Sayal K and Hollis C (2013) Transition to adult mental health services for young people with attention deficit/hyperactivity disorder (ADHD): A qualitative analysis of their experiences. *BMC Psychiatry* 13, 74. 10.1186/1471-244X-13-74 PMC3605266 23497082

[ref27] van Staa A, Peeters M and Sattoe J (2020) On your own feet: A practical framework for improving transitional care and young people’s self-management. In Betz CL and Coyne IT (eds), *Transition from Pediatric to Adult Healthcare Services for Adolescents and Young Adults with Long-term Conditions*. Cham: Springer International Publishing, 191–228.

[ref28] White PH, Cooley WC, Boudreau ADA, Cyr M, Davis BE, Dreyfus DE, Forlenza E, Friedland A, Greenlee C, Mann M, McManus M, Meleis AI and Pickler L (2018) Supporting the health care transition from adolescence to adulthood in the medical home. *Pediatrics* 142, e20182587. 10.1542/peds.2018-2587 30348754

[ref29] Young S, Murphy CM and Coghill D (2011) Avoiding the ‘twilight zone’: Recommendations for the transition of services from adolescence to adulthood for young people with ADHD. *BMC Psychiatry* 11, 174. 10.1186/1471-244X-11-174 PMC3229466 22051192

