# Supplementary material for: Challenges in transitioning from adolescent to Adult Mental Health Services for young adults with ADHD in Italy: an observational study
Source: Epidemiol Psychiatr Sci. 2024 Oct 24;33:e49. doi: 10.1017/S2045796024000544 (PMC11561684; doi:10.1017/S2045796024000544)
Supplement: Roberti et al. supplementary material 2 — Roberti et al. supplementary material [file S2045796024000544sup002.pdf]

ID	At age 18 services	Intermediate period other services	Current services	referred	outcome/reason for unsuccessful referral	impairment	need for support	employment	sentinel events	medications before transfer	current medications
2	AMHS		AMHS	-	*already cared for by AMHS at 18*	moderate	no	unemployed		drugs + therapy	no treatment
3	CAMHS + AMHS	CAMHS + AMHS	AMHS	-	*already cared for by AMHS at 18*	moderate	no	school		drugs + therapy	no treatment
4	AMHS		AMHS	-	*already cared for by AMHS at 18*	moderate	yes	school		drugs + therapy	no treatment
5	AMHS	none	none	-	*already cared for by AMHS at 18*	moderate	no	school		drugs + therapy	drugs + therapy
7	CAMHS	AMHS + private	AMHS	yes	*referral successful*	moderate	no	unemployed		drugs + therapy	no treatment
21	CAMHS	AMHS + private	AMHS +private +center for drug addiction	no	lack of specialized service	moderate	yes	unemployed		drugs + therapy	no treatment
22	CAMHS	private	CAMHS only for medications	no	deemed unnecessary	mild	no	school		drugs + therapy	no treatment
23	CAMHS	AMHS	AMHS	yes	*referral successful*	medium/severe	no	unemployed		drugs + therapy	no treatment
8	CAMHS	AMHS	AMHS	yes	*referral successful*	mild	yes	unemployed		drugs + therapy	therapy
11	CAMHS	none	AMHS + private	yes	*referral successful*	mild	yes	work+school	yes	drugs	no treatment
36	CAMHS	CAMHS	territorial CAMHS	yes	*referral successful but not to AMHS*	moderate	yes	work		drugs + therapy	therapy
17	CAMHS	none	none	yes	dropout, no compliance	moderate	no	work		no treatment	therapy
18	CAMHS	none	none	no	lack of specialized service	mild	yes	work		drugs + therapy	no treatment
19	CAMHS	none	none	no	lack of specialized service	moderate	yes	work	yes	drugs + therapy	therapy
20	CAMHS	none	none	no	lack of specialized service	moderate	no	work		drugs + therapy	no treatment
24	CAMHS	none	none	no	dropout, no compliance	medium/severe	no	work		drugs + therapy	no treatment
9	CAMHS	none	none	no	lack of specialized service	mild	yes	school		drugs + therapy	no treatment
10	CAMHS	none	none	no	other CAMHS for complex clinical picture	moderate	no	work		no treatment	drugs
12	CAMHS	none	none	yes	*referral successful*	mild	no	unemployed		drugs	drugs + therapy
13	CAMHS	none	none	yes	*referral successful*	mild	yes	work+school	yes	drugs + therapy	drugs + therapy
14	CAMHS	none	none	no	lack of specialized service	moderate	yes	unemployed	yes	drugs + therapy	no treatment
15	CAMHS	none	none	no	lack of specialized service	moderate	no	work	yes	therapy	drugs + therapy
29	private	AMHS	private	no	dropout, no compliance	medium/severe	yes	work		drugs + therapy	no treatment
30	private	AMHS + private	private	yes*	attempted but unsuccessful	medium/severe	no	school		drugs + therapy	no treatment
6	private	none	none	yes*	attempted but unsuccessful	moderate	yes	school		therapy	drugs
31	private	PRIVATE	private	yes*	attempted but unsuccessful	medium/severe	yes	work+school		drugs + therapy	no treatment
25	private	none	none	no	patient chose a private professional	mild	no	work		drugs + therapy	therapy
1	none	AMHS	AMHS	yes	attempted but unsuccessful	medium/severe	yes	school		drugs + therapy	drugs
16	none	private	none	no	lack of specialized service	mild	yes	unemployed		drugs + therapy	no treatment
33	none	none	none	no	deemed unnecessary	mild	no	work	yes	drugs + therapy	no treatment
34	none	none	none	no	deemed unnecessary	medium/severe	no	work	yes	drugs + therapy	no treatment
35	none	none	none	no	deemed unnecessary	mild	no	work		drugs + therapy	no treatment
32	none	none	none	no	deemed unnecessary	mild	no	work		drugs + therapy	no treatment
26	none	none	none	no	deemed unnecessary	mild	no	work		drugs + therapy	no treatment
27	none	none	none	no	deemed unnecessary	mild	no	work	yes	drugs + therapy	no treatment
28	none	none	none	no	dropout, no compliance	mild	no	work		drugs + therapy	no treatment

Notes: "Intermediate period" refers to the time between young adults turning 18 and the year before the interview.

"Current" refers to the time of the interview and the year before the interview

* referral only attempted
